# Epistatic Adaptive Evolution of Human Color Vision

**DOI:** 10.1371/journal.pgen.1004884

**Published:** 2014-12-18

**Authors:** Shozo Yokoyama, Jinyi Xing, Yang Liu, Davide Faggionato, Ahmet Altun, William T. Starmer

**Affiliations:** 1 Department of Biology, Emory University, Atlanta, Georgia, United States of America; 2 Department of Biology, Emory University, Atlanta, Georgia, United States of America; 3 College of Life Science, Linyi University, Linyi, Shandong, China; 4 Department of Physics, Fatih University, Istanbul, Turkey; 5 Department of Biology, Syracuse University, Syracuse, New York, United States of America; University of Michigan, United States of America

## Abstract

Establishing genotype-phenotype relationship is the key to understand the molecular mechanism of phenotypic adaptation. This initial step may be untangled by analyzing appropriate ancestral molecules, but it is a daunting task to recapitulate the evolution of non-additive (epistatic) interactions of amino acids and function of a protein separately. To adapt to the ultraviolet (UV)-free retinal environment, the short wavelength-sensitive (SWS1) visual pigment in human (human S1) switched from detecting UV to absorbing blue light during the last 90 million years. Mutagenesis experiments of the UV-sensitive pigment in the Boreoeutherian ancestor show that the blue-sensitivity was achieved by seven mutations. The experimental and quantum chemical analyses show that 4,008 of all 5,040 possible evolutionary trajectories are terminated prematurely by containing a dehydrated nonfunctional pigment. Phylogenetic analysis further suggests that human ancestors achieved the blue-sensitivity gradually and almost exclusively by epistasis. When the final stage of spectral tuning of human S1 was underway 45–30 million years ago, the middle and long wavelength-sensitive (MWS/LWS) pigments appeared and so-called trichromatic color vision was established by interprotein epistasis. The adaptive evolution of human S1 differs dramatically from orthologous pigments with a major mutational effect used in achieving blue-sensitivity in a fish and several mammalian species and in regaining UV vision in birds. These observations imply that the mechanisms of epistatic interactions must be understood by studying various orthologues in different species that have adapted to various ecological and physiological environments.

## Author Summary

Mapping the genotype-phenotype relationship is necessary to understand how variable phenotypes have evolved in nature. The blue-sensitive visual pigment in human (human S1) evolved from the UV-sensitive pigment in the Boreoeutherian (or Boreotherian) ancestor (AncBoreotheria S1) by seven mutations. Mutagenesis experiments reveal that 4,008 (∼80%) of all 5,040 possible evolutionary trajectories connecting from AncBoreotheria S1 to human S1 are terminated prematurely. Quantum chemical analyses suggest that the premature termination of trajectories was caused by containing a dehydrated nonfunctional pigment. Phylogenetic analysis further suggests that the blue-sensitivity was achieved only gradually and almost exclusively by the seven non-additively interacting amino acids. During the period between 45 and 30 My ago, human S1 was in the final stage of developing its blue-sensitivity. This was the time when two red-sensitive pigments appeared by gene duplication and one of them became green-sensitive. Trichromatic color vision in the human lineage was fully developed by 30 My ago by interprotein epistasis among the three visual pigments. Manipulation of the genetically engineered ancestral molecule is the key to recapitulate the evolution of phenotypic adaptation and that of epistatic interaction separately.

## Introduction

The chance of survival of novel mutations is affected strongly by the molecular background in which they appear [1]–[3]. To understand the evolutionary dynamics of these mutations, it is necessary to characterize the phenotypic variation they generate. For vertebrates, however, understanding the genotype-phenotype relationship remains challenging because of technical difficulties in connecting the structure and function of evolving proteins and in evaluating non-additive (epistatic) interactions among amino acids unambiguously [3]–[7].

About 90% of human populations detect the entire range of visible color using three types of cone pigments: short wavelength-sensitive (SWS1) pigment (human S1), middle wavelength-sensitive (MWS) pigment (human M) and long wavelength-sensitive (LWS) pigment (human L), which detect light maximally (λ_max_) at 414, 530 and 560 nm, respectively [8], [9]. Human S1 can be made UV-sensitive (λ_max_ = 360 nm) by introducing seven mutations T46F, L49F, F52T, L86F, P93T, G114A and T118S, whereas the UV-sensitive pigment in mouse (*Mus musculus*) (mouse S1, λ_max_ = 359 nm) can be made blue-sensitive (λ_max_ = 411 nm) by the seven reverse mutations; however, when the seven mutations are introduced into mouse S1 individually, none of the individual changes produce any λ_max_-shift [10], showing an extreme case of epistatic interactions. Largely free from the technical difficulties in evaluating the genotype-phenotype relationships as well as their strong associations to variable ecological and physiological environments, visual pigments make vertebrate vision a powerful model to directly study the dynamics of genotype-phenotype relationship during phenotypic adaptation [11]–[13]. The crystal structure of the visual pigments in bovine rod photoreceptors [14], [15] and a large dataset on ecology of vision [16]-[18] also allow us to link the chemistry, genetics, organismal biology and ecology of vertebrate vision.

Regulated primarily by the lens [19], photons with wavelengths shorter than 400 nm do not reach the retinas of some primates, including human, and sciurid rodents, whereas UV light does reach the retinas of mouse, rat and other mammals [19], [20]. As it may be suspected from these observations, SWS1 pigments in most vertebrate ancestors, including the mammalian ancestor, had λ_max_ values of 360 nm and were UV-sensitive [21]. Subsequently, in the lineage leading to humans, the UV-transmitting lens evolved into a UV-absorbing lens [20]. To adapt to the UV-free retinal environment [19], [20], [22], human S1 switched from detecting UV to absorbing blue light during the last 90 million years (My) [21].

Making the UV-sensitive mouse S1 blue-sensitive may give an impression that human S1 evolved from the SWS1 pigment of the Boreoeutherian (or Boreotherian) ancestor (AncBoreotheria S1) by F46T, F49L, T52F, F86L, T93P, A114G and S118T. To prove this, however, we have to show that these mutations actually switched the λ_max_ of AncBoreotheria S1 into that of human S1. Even if such critical mutations are identified, it is still unclear how they have accumulated mutations and modified the λ_max_ of AncBoreotheria S1 during evolution. To address these questions, we engineer AncBoreotheria S1, construct all possible evolutionary trajectories that connect AncBoreotheria S1 and human S1, recapitulate the evolutionary changes in the epistatic interaction and the λ_max_ separately; in this way, we establish the genotype-phenotype relationship unambiguously during the entire process of human S1 evolution. We then consider how human ancestors acquired the present trichromatic color vision based on the three cone pigments.

## Results

### Evolutionary trajectories

**Figure 1:**
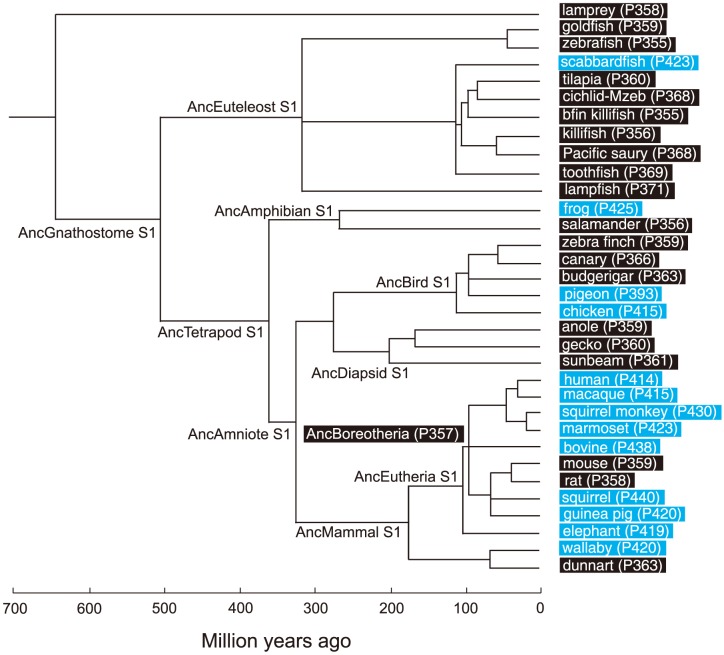
A phylogenetic tree of 33 representative SWS1 pigments. The numbers after P indicate the λ_max_ values. Divergence times inferred using through the “TimeTree of Life” web server (www.Timetree.org) are shown at the bottom. Black and blue rectangles indicate UV- and blue-sensitive pigments, respectively.

**Figure 2:**
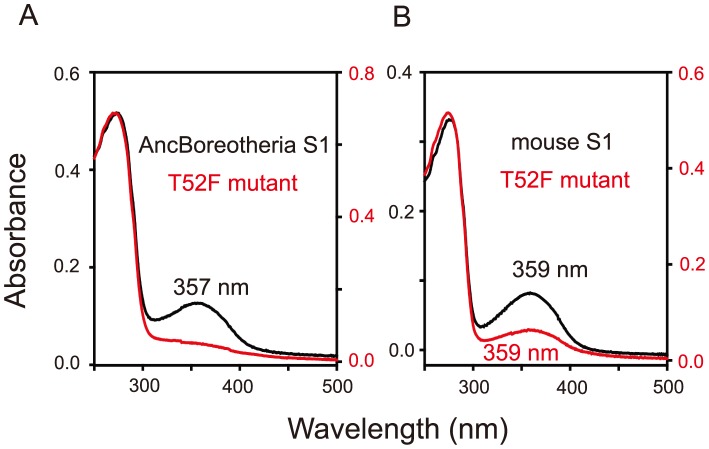
Absorption spectra of SWS1 pigments. (A) AncBoreotheria S1 and T52F mutants. AncBoreotheria S1 has a λ_max_ of 357 nm and is UV-sensitive, whereas the T52F mutant does not form a functional visual pigment. (B) Mouse S1 and T52F mutants. Both pigments have λ_max_ values of 359 nm and are UV-sensitive. All of these pigments were regenerated by incubating the opsins with 11-*cis*-retinal (a gift from Dr. Rosalie K. Crouch and the National Eye Institute) and were purified using immobilized 1D4 (The Culture Center, Minneapolis, MN). UV visible spectra were recorded at 20°C using a Hitachi U-3000 dual beam spectrophotometer. Visual pigments were bleached for 3 min using a 60 W standard light bulb equipped with a Kodak Wratten #3 filter at a distance of 20 cm. Data were analyzed using Sigmaplot software (Jandel Scientific, San Rafael, CA).

Based on the phylogenetic tree of 33 representative SWS1 pigments sampled from a wide range of vertebrate species (Fig. 1), the amino acid sequences at different nodes have been inferred using PAML [23] and AncBoreotheria S1 was reconstructed (for details, see Methods). The *in vitro* assay shows that AncBoreotheria S1 has a λ_max_ (or simply λ) of 357 nm (Fig. 2A, in black spectrum) and its mutant with F46T, F49L, T52F, F86L, T93P, A114G and S118T has a λ_max_ (λ_F46T/F49L/T52F/F86L/T93P/A114G/S118T_) of 411 nm (S1 Table). These results, indeed, demonstrate that human S1 evolved from AncBoreotheria S1 (or AncBoreotheria (P357)) by accumulating the seven mutations.

To accommodate seven sequential amino acid replacements, there are 7! ( = 5,040) possible evolutionary trajectories. We traced all these trajectories by introducing seven single and all 120 possible combinations of the seven mutations into AncBoreotheria S1 and evaluating their effects on the λ_max_-shift (or Δλ_max_). The *in vitro* assays revealed that 60 out of 127 mutants could not make functional visual pigments, making 4,008 possible evolutionary paths prematurely terminated (S1 Fig.). These incomplete trajectories are caused most often by T52F primarily because if this mutation occurs first, the evolutionary path is immediately terminated. The phenotypic difference between AncBoreotheria S1 and the T52F mutant is obvious; that is, compared with the absorption spectrum of AncBoreotheria S1 with a clear peak at 357 nm, the T52F mutant does not have any absorption peak for the entire region of visible light, showing that the mutant is structurally unstable (Fig. 2A, in red spectrum). If F46T, F49L, F86L, T93P, A114G or S118T occur first, the numbers of trajectories that can accumulate the remaining 6 mutations are 134, 74, 252, 348, 102 and 122, respectively, with various magnitudes of Δλ_max_ values during evolution (S2 Fig.). Therefore, a total of 1,032 (20.47%) out of the 5,040 possible trajectories are evolutionarily accessible.

### Quantum chemistry of T52F

Interestingly, mouse S1 has a λ_max_ of 359 nm and is UV-sensitive like AncBoreotheria S1 (Fig. 2B, in black spectrum), but its T52F mutant is functional (Fig. 2B, in red spectrum) [10]. At the chemical level, each visual pigment consists of a mixture of pigments with protonated Schiff base nitrogen-linked 11-*cis*-retinals (PSBR) and those with unprotonated Schiff base nitrogen-linked 11-*cis*-retinals (SBR) [24]. An SWS1 pigment is UV-sensitive when SBR is more stable than PSBR; otherwise it is blue-sensitive. Moreover, the relative stability of a pigment with SBR and PSBR depends strongly on the water molecules around the 11-*cis*-retinal [24]–[26]. The cause for the contrasting roles of T52F in AncBoreotheria S1 and mouse S1 can be seen in two steps.

**Figure 3:**
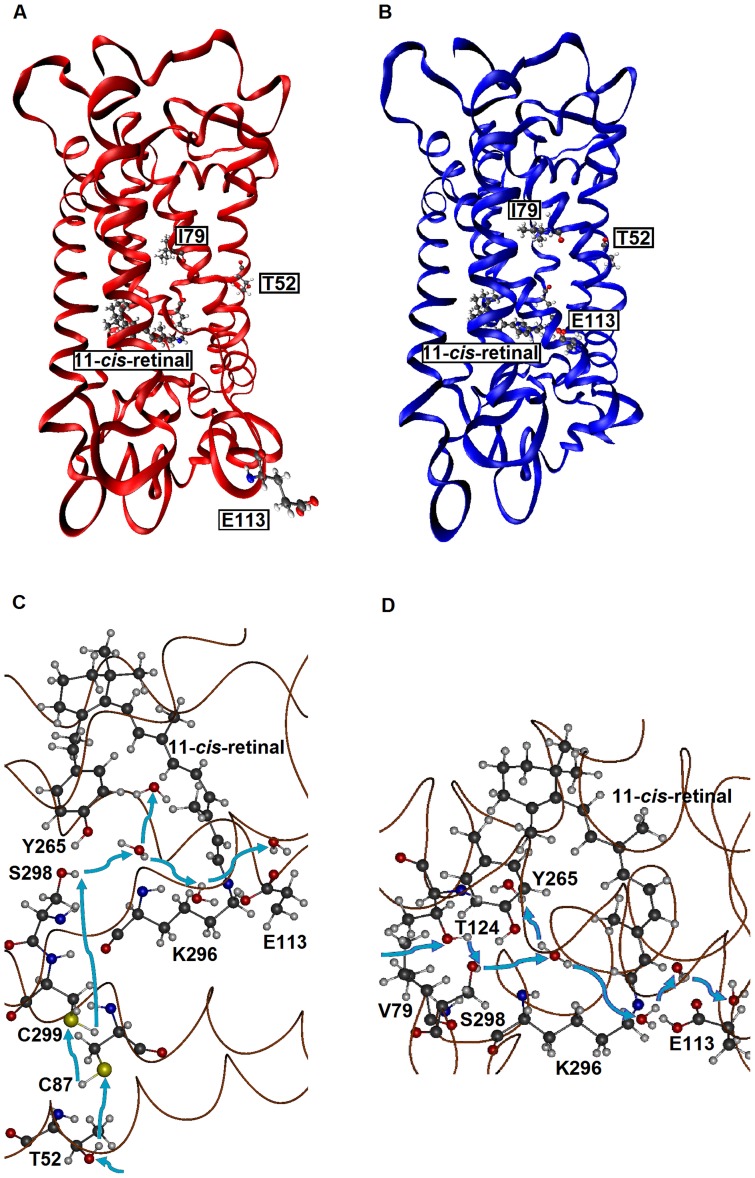
The tertiary structures of SWS1 pigments. (A) A dehydrated model of AncBoreotheria S1 with SBR and protonated E113, which is located at the protein surface. (B) A hydrated model of AncBoreotheria S1 with PSBR, where E113 is located next the 11-*cis*-retinal. (C) Water channel connected to T52 of mouse S1. (D) Channel connected to V79 of mouse S1. Blue arrows indicate the directions of water movement. Black, blue, red and white molecules represent carbon, nitrogen, oxygen and hydrogen atoms, respectively. The structures of AncBoreotheria S1 and mouse S1 pigments were obtained by 1) applying homology modeling (Modeller 9v7, www.salilab.org/modeller) to bovine rhodopsin (pdb code: 1U19), 2) adding the missing hydrogen atoms, water molecules and 11-*cis*-retinal, and 3) optimizing them first at pure AMBER96 force field level (http://ambermd.org) and then using hybrid quantum mechanical/molecular mechanical (QM/MM) calculations in the ONIOM electronic embedding scheme (QM  =  B3LYP/6–31G*; MM  =  AMBER).

First, when they are hydrated, AncBoreotheria S1, mouse S1, and their T52F mutants with SBR are all 7 kcal/mol more stable than their PSBR counterparts, which show that all of these pigments are UV-sensitive. In the dehydrated states, the pigments with PSBR achieve similar H-bond interactions; much to our surprise, however, the pigments with SBR become nonfunctional because E113 moves to the protein surface (Fig. 3A). To attain a functional pigment, therefore, water molecules that keep E113 near the 11-*cis*-retinal are required (Fig. 3B). Second, mouse S1 and AncBoreotheria S1 consist of different types of water channels that allow water molecules to flow from the surface to the interior of the pigments: mouse S1 has two surface openings at T52 and V79 (Figs. 3C, D), but AncBoreotheria S1 has basically one opening at site 52 because of bulky I79. When T52F is introduced into AncBoreotheria S1, the bulky F52 blocks its only water channel; however, in mouse S1 with T52F, the channel opening through V79 is still operational. Hence, the mouse S1 mutant can still be hydrated, but the AncBoreotheria S1 mutant becomes dehydrated and is nonfunctional. In general, such disruptions in water trafficking affecting the H-bond network [24] near the 11-*cis*-retinal seem to be a major cause for generating nonfunctional pigments.

### The mode of phenotypic adaptation of human S1

**Figure 4:**
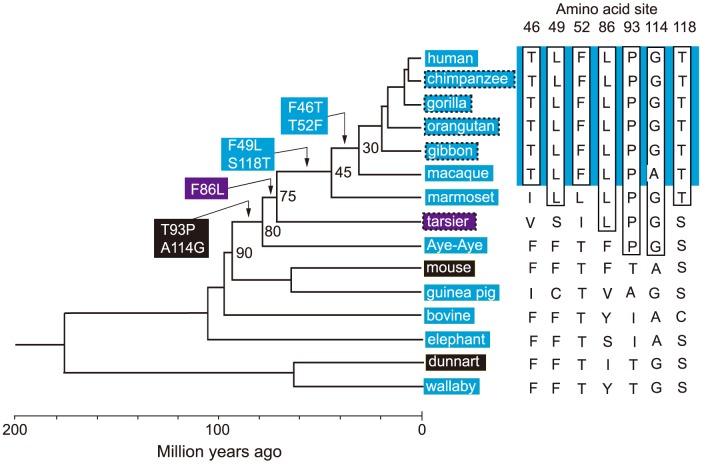
Patterns of amino acid replacements in human S1. The evolutionary tree of representative mammalian SWS1 pigments, where black, blue and purple rectangles indicate UV-, blue- and their intermediate color-sensitive pigments, respectively (left panel) and their amino acid compositions (right panel). The rectangles surrounded by broken lines indicate their suspected color sensitivities. Amino acids in rectangle (right panel) indicate that they occurred once at that site, where the identical amino acid compositions of the primate pigments are highlighted by blue color and the four steps of amino acid replacements have been inferred from them (left panel). The numbers at different nodes indicate the divergence times, which have been estimated previously [27], [28].

**Figure 5:**
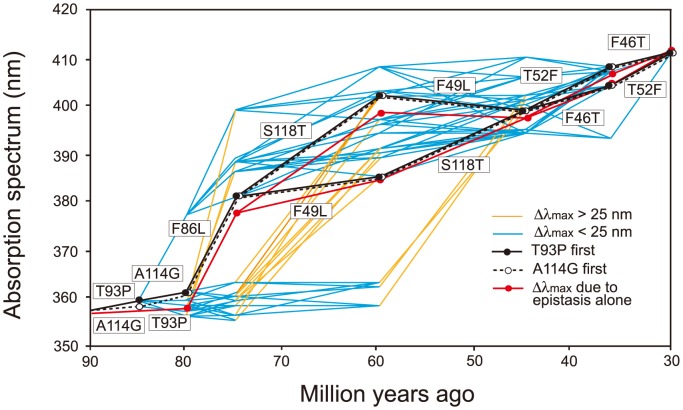
Eight most likely evolutionary paths used during the evolution of human S1. Human S1 evolution starting either with T93P (in black lines) or with A114G (in broken lines) are shown separately. In the background, a total of 442 evolutionarily accessible trajectories are given. The eight trajectories are characterized by the gradual increase in their λ_max_ values, consisting of each step with |Δλ_max_|<25 nm. The path in red shows the λ_max_ values predicted by considering only epistatic interactions among the seven mutations. Δλ_max_ values smaller (in blue) or larger (in orange) than 25 nm are distinguished.

It would be ideal if we could identify the evolutionary trajectory that actually took place in the evolution of human S1. At present, we can consider the SWS1 pigments of nine primate species for this purpose. A composite evolutionary tree of these pigments and those of six other mammalian species reveals that 1) T93P and A114G, 2) F86L, 3) F49L and S118T and 4) F46T and T52F occurred in that order. Hence, we can identify eight most likely trajectories for describing human S1 evolution (Fig. 4). Then, going back to our mutagenesis results and using the divergence times estimated by others [27], [28], we can see that the ancestral human S1 remained UV-sensitive until about 80 My ago and its λ_max_ increased 20 nm in the next 5 My and another 20 nm in the next 30 My, thus reaching 400 nm by 45 My ago and, finally, the current λ_max_ value was achieved by 30 My ago (Fig. 5, in black and broken trajectories).

When the seven critical amino acid replacements are started with T93P or A114G, the number of trajectories total 450. The eight most likely trajectories are clustered in the center of the 450 trajectories and are characterized by the gradual increase in their λ_max_ values, each step consisting with |Δλ_max_|<25 nm. Further support for this conclusion comes from an analysis of the relatively smaller variance for the amount of change for pathways predicted by the phylogeny as compared with the distribution of all possible variances (S3 Fig.). The gradual λ_max_-shifts might have been necessary because our ancestors switched from their nocturnal life to a diurnal-life style by adjusting their vision slowly to various twilight conditions. The slowly evolving human S1 implies that the evolutionarily acceptable 1,032 trajectories can be subdivided further depending on whether they are characterized by |Δλ_max_|<25 nm at every evolutionary step (335 paths, 32.5%) or not (67.5%). In this sense, the evolutionary trajectory of human S1 might have been any one of the 335 evolutionary trajectories (6.65% of 5,040 possible trajectories).

### The molecular basis of the phenotypic adaptation of human S1

Knowing the λ_max_ of AncBoreotheria S1 and those of all 127 mutant pigments allows us to evaluate the Δλ_max_ values caused by F46T (θ_46_)… S118T (θ_118_) as well as those of two-way (θ_46×49_, θ_46×52_… θ_114×118_)… and seven-way (θ_46×49×52×86×93×114×118_) interactions. To follow this method, it becomes necessary to infer the λ_max_ values of 60 nonfunctional pigments. This is possible because when a new mutation prevents the formation of a functional pigment or when it actually does not shift the λ_max_, the highest level of an epistatic effect can be regarded as zero and the λ_max_ of the mutant pigment can be estimated (see Statistical Analyses, Methods, S1 Table; unstable in bold italics). One special example of this is θ_52_  = 0, which was established using the mouse T52F mutant (Fig. 2B). This procedure is required if we want to obtain all 120 epistatic interaction terms; but, as we will see below, the λ_max_-shift of each evolutionary path can be recapitulated without obtaining all individual θ values.

**Figure 6:**
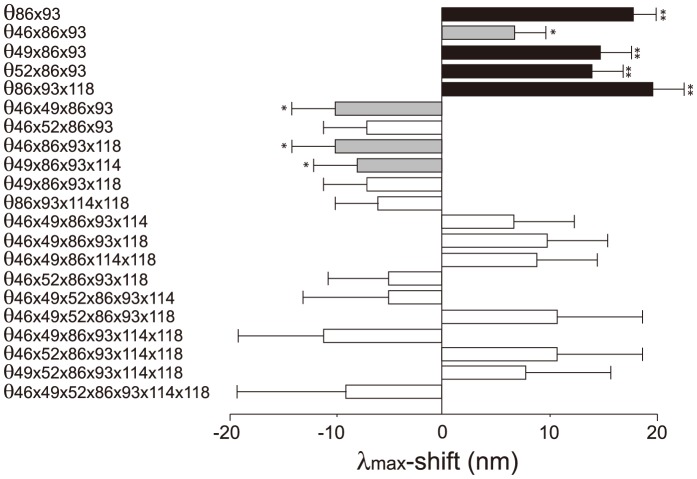
The θ values (|θ|>5 nm) that are generated by epistasis in AncBoreotheria S1. The λ_max_ values of the 127 SWS1 mutant pigments were expressed as that of AncBoreotheria S1 (λ) plus the effects of the appropriate single and multiple amino acid changes on the λ_max_-shift (denoted by a sum of θ values). These θ values were estimated by solving a total of 128 simultaneous linear equations with the ancestral λ and 127 mutant values. The individual effects of θ_46_, θ_49_, θ_52_, θ_86_, θ_93_, θ_114_ and θ_118_ are −2, −3, 0, 0, 2, 1 and 1 nm, respectively, and their roles in human S1 evolution are negligible compared with those of epistatic interactions. The graph shows the 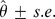 *P<0.05. **P<0.01.

The 127 θ values determined from 127 linear equations show two things. First, the individual mutational effects on the Δλ_max_ are very close to zero, i. e. θ_46_ = −2, θ_49_ = −3, θ_52_ = θ_86_ = 0, θ_93_ = 2, θ_114_ = θ_118_ = 1 (S2 Table). Second, 21 out of 120 interaction terms show that |θ| ≥5 nm, most of which (20 out of 21) reflect significant influences of the interaction between F86L and T93P; in particular, F86L is always involved in generating measurable epistatic interactions (Fig. 6). Consequently, the λ_max_ values for the eight most likely trajectories are explained mostly by the epistatic interactions alone (Fig. 5, in red trajectories). As it was indicated earlier, the evolution of the λ_max_ of human S1 during the last 90 My can be recapitulated without estimating all θ values individually. For example, when we consider the trajectory with the most conservative functional change, i. e. the smallest |Δλ_max_| values, each step of the observed phenotypic change can be explained solely by epistatic effects (S3 Table).

## Discussion

Human S1 evolved gradually from AncBoreotheria S1. The blue-sensitive pigment in African clawed frog (*Xenopus laevis*) (frog S1, λ_max_ = 425 nm) also evolved from the UV-sensitive pigment of the Amphibian ancestor (AncAmphibian S1, λ_max_ = 359 nm; Fig. 1) by accumulating F86M, V91I, T93P, V109A, E113D, L116V and S118T [29]. Again, because of their small individual mutational effects on the λ_max_-shift [29], the phenotypic evolution of frog S1 occurred mostly by epistatic interactions. Living in shallow waters, the light environments of the frog ancestors must have been similar to those of human ancestors. Hence, it is likely that the transition from the λ_max_ of AncAmphibian S1 to that of frog S1 occurred gradually.

SWS1 pigments can also take dramatically different modes of evolution. For example, the blue-sensitive pigment in scabbardfish (*Lepidopus fitchi*) (scabbardfish S1, λ_max_ = 423 nm) evolved from the UV-sensitive pigment in the euteleost ancestor (AncEuteleost S1, Fig. 1) by the deletion of F86 [26]. Similarly, the blue-sensitive pigments in bovine (*Bos taurus*) and wallaby (*Macropus eugenii*) also evolved essentially in one step by single mutations F86Y [5], [30], [31]. In addition, C90S makes the UV-sensitive pigments of zebra finch (*Taeniopygia guttata*) and budgerigar (*Melopsittacus undulates*) blue-sensitive [32], [33], whereas S90C transforms the blue-sensitive pigments of chicken (*Gallus gallus*) and pigeon (*Columba livia*) into UV-sensitive pigment [32].

These observations seem to suggest that there are two distinct modes of evolution among SWS1 pigments. However, even when major λ_max_-shifts are caused by single mutations, epistatic interactions cannot be ignored. For example, the deletion of F86 in AncEuteleost S1 seems to increase the λ_max_ by 59 nm, but the same mutation in the UV-sensitive pigment in a vertebrate ancestor (AncGnathostome S1, λ_max_ = 360 nm; Fig. 1) increases the λ_max_ only by 19 nm [26]. Hence, despite the same UV-sensitivity of the two ancestral pigments, their molecular backgrounds are critically different, causing different epistatic effects on the λ_max_-shift. Similarly, S90C causes a wide range of λ_max_-shifts, between -46 nm and 0 nm, depending on SWS1 pigments manipulated [10], [21], [31]–[34].

During the period between 45 and 30 My ago, the last two amino acid replacements (F46T and T52F) were in progress in the ancestral human S1 pigment (Fig. 5). This was the time when the LWS pigment in the Boreotherian ancestor (or AncBoreotheria L) achieved two critical changes. First, two AncBoreotheria L copies were generated by a gene duplication event and, second, one of them retained the ancestral λ_max_ (560 nm) and became modern human L and the other decreased its λ_max_ to 530 nm by accumulating three mutations (S180A, Y277F and T285A) and became the modern human M [35]–[37]. At present, the order of S180A, Y277F and T285A in AncBoreotheria L is not known. However, mutagenesis analyses of AncBoreotheria L show that θ_180_ = −5, θ_277_ = −10, θ_285_ = −17, θ_180×277_ = 0, θ_180×285_ = −2, θ_277×285_ = 1 and θ_180×277×285_ = 4 and the sum of these values is −29 nm, which fully explains the evolution of human M [36]. Hence, if S180A and Y277F occurred before T285A, the λ_max_ of the mutant pigment was 545 nm; on the other hand, if T285A was included in the first two mutations, the λ_max_ values of the mutants were 532–538 nm, much similar to that of human M.

**Figure 7:**
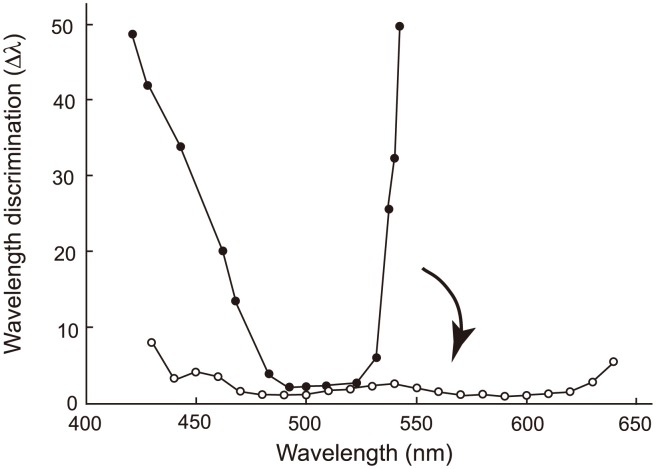
Evolutionary change in the wavelength discrimination by human ancestors. Forty-five My ago, our ancestor possessed almost final product of human S1 and human L and could have achieved color vision of deuteranopes who do not have functional MWS pigment (represented by a discrimination function with black circles). This has changed to the color vision of trichromats with the three cone pigments (represented by a discrimination function with white circles) in the following 15 My. The arrow indicates this evolutionary change. The wavelength discrimination functions of deuteranope and trichromat data are those of A. W. G. in [48] and W. D. W. in [49], respectively.

Eventually, we are interested in another phenotype that is synthesized by human S1, human M and human L – color vision. Color vision may be best characterized by wavelength discrimination, which describes the minimum wavelength difference (Δλ) along a wide range of wavelengths that human, or any experimental animals, can discriminate [13], [17], [38]. A typical trichromat having the three cone pigments exhibits Δλ<3 nm, sometimes Δλ<1 nm, along the wavelength between 450 and 625 nm (Fig. 7, Δλwith white circles); in contrast, deuteranopes or protanopes, who are missing functional human M or human L, respectively, have Δλ<5 nm only at around 500 nm (Fig. 7, Δλ with black circles for a deuteranope) [17], [39]. Incorporating one or two of the three critical amino acids into AncBoreotheria L, our ancestors have achieved different levels of anomalous trichromatic color vision, conditions known as deuteranomaly, who achieve intermediate wavelength discrimination functions between those of deuteranopes and trichromats. That is, deuteranomalous people can discriminate the wavelength outsides of 500 nm, particularly at around 600 nm, much better than deuteranopes (e.g. [40]). Therefore, as the three critical mutations accumulate in one of the duplicated AncBoreotheria L pigments, the U-shaped discrimination function of the human ancestor started to become more flat and eventually reached the more flat discrimination function of modern trichromats (Fig. 7).

The evolution of human S1 could have taken any one of a small proportion of trajectories (335 out of 5,040 possible trajectories, or 6.65%). This observation is consistent with the results of epistatic adaptive evolutionary studies such as antibiotic resistance [2], drug resistance [41], coenzyme evolution [2], [42] and coevolution of two ecotypes [43] in microbial systems as well as the evolution of hormone receptors [7], visual pigments [44] and hemoglobins [45] in vertebrates. However, one important implication of epistasis, often neglected, is that forward and reverse mutations can depict dramatically different epistatic interactions, leading us to erroneous conclusions on the mechanisms of phenotypic adaptation [4], [5]. To obtain the correct molecular mechanism of phenotypic adaptation, therefore, it is critical not only to identify appropriate ancestral molecules, engineer and manipulate them but also to recapitulate the evolution of epistatic interactions.

## Materials and Methods

### Ethics statement

The project does not involve any live animals and has been approved by the Institutional Animal Care and Use Committee of the Emory University, in compliance with the USA Public Health Service Policy on Human Care and Use of Laboratory Animals.

### Reconstruction of the ancestral SWS1 pigment

To infer the amino acid sequences at various ancestral pigments, we have constructed a composite phylogenetic tree of 33 representative SWS1 pigments (Fig. 1). Using these sequences and those of RH1 pigment of bovine (*Bos Taurus*; M21606), RH2 pigment of goldfish (*Carassius auratus*; L11865) and SWS2 pigment of zebrafish (*Danio rerio*; AB087809) as the outgroup, we inferred the amino acid sequences of SWS1 pigments at various nodes of the phylogenetic tree using PAML [23]. The SWS1 pigments of 33 vertebrate species have been considered were as follows: lamprey (*Lamptera marinus*; U67123), goldfish (*C. auratus*; D85863), zebrafish (*D. rerio*; AB087810), scabbardfish (*Lepidopus fitchi*; FJ443126), tilapia (*Oreochromis niloticus*; AF191221), cichlid-Mzeb (*Maylandia zebra*; AF191219), bluefin killifish (*Lucania goodei*; AY296735), medaka (*Oryzias latipes*; AB223058), Pacific saury (*Cololabis saira*; KP099197), toothfish (*Dissostichus mawsoni*; AY927651), lampfish (*Stenobrachius leucepsarus*; FJ443127), frog (*Xenopus laevis*; U23463), salamander (*Ambystoma tigrinum*; AF038948), zebra finch (*Taeniopygia guttata*; AF222331), canary (*Serinus canaria*; AJ277922), budgerigar (*Melopsittacus undulatus*; Y11787), pigeon (*Columba livia*; AF149234), chicken (*Gallus gallus*; M92039), anole (*Anolis carolinensis*; AF134192), gecko (*Gekko gekko*; AY024356), sunbeam (*Xenopeltis unicolor*; FJ497234), human (*Homo sapiens*; M13295), macaque (*Macaca fascicularis*; AF158977), squirrel monkey (*Saimiri sciureus*; U53875), marmoset (*Callithrix jacchus*; L76201), Bovine (*Bos taurus*; U92557), mouse (*Mus musculus*; U49720), rat (*Rattus norvegicus*; U63972), squirrel (*Sciurus carolinensis*; DQ302163), guinea pig (*Cavia porcellus*; AY552608), elephant (*Loxodonta africana*; AY686753), wallaby (*Macropus eugenii*; AY286017) and dunnart (*Sminthopsis crassicaudata*; AY442173).

AncBoreotheria S1 differs from human S1 and the previously engineered ancestral mammalian pigment (denoted as pigment g) considering a much smaller number of SWS1 pigments [21], at 25 (S4 Fig.) and 9 amino acid sites, respectively. Hence, we reconstructed AncBoreotheria S1 by introducing a total of 9 amino acid changes into pigment g. It should be noted that the N terminus (sites 1–30) and C terminus (sites 313–348) of pigment g and AncBoreotheria S1 are taken from those of the SWS1 pigment of *Anolis carolinensis* (anole S1). This is done to standardize the effects of variable amino acids at the N- and C-termini of SWS1 pigments on the λ_max_-shift. This procedure is justified because when the two segments of mouse S1 are replaced by those of human S1, anole S1 and the orthologous goldfish pigment, their λ_max_ values are 359–360 nm, showing that the sequence variation in the N and C termini do not affect the spectral tuning in SWS1 pigments [21].

Mutant opsins were generated by using QuickChange site-directed mutagenesis kits (Stratagene, La Jolla, CA). To rule out spurious mutations, the DNA fragment was sequenced by cycle sequencing reactions using the Sequitherm Excel II long-read kits (Epicentre Technologies, Madison, WI) with dye-labeled M13 forward and reverse primers. Reactions were run on a LI-COR (Lincoln, NE) 4300LD automated DNA sequencer.

To consider the phylogeny of 15 mammalian species, the SWS1 pigments of six additional species have been included: chimpanzee (*Pan troglodytes*; AF039433), gorilla (*Gorilla gorilla gorilla*; XM_004046176), orangutan (*Pongo abelii*; XM_002818421), gibbon (*Nomascus leucogenys*; XM_003261297), tarsier (*Tarsius bancanus*; AB111463) and Aye-Aye (*Daubentonia madagascariensis*; EF667285). TimeTree of Life web server (www.timetree.org) shows that human diverged from macaque, marmoset, tarsier, Aye-Aye and mouse about 30, 45, 75, 80 and 90 My ago, respectively [27], [28]. Then, following the parsimony assumption that minimizes the number of amino acid replacements, the possible evolutionary pathways for the seven amino acid replacements have been determined.

### The *in vitro* assay

The opsin cDNA clones were expressed in COS1 cells by transient transfection [46]. The pigments were regenerated by incubating the opsins with 11-*cis*-retinal (a gift from Dr. Rosalie K. Crouch at Storm Eye Institute, Medical University of South Carolina and National Eye Institutes) and were purified using immobilized 1D4 (The Culture Center, Minneapolis, MN) in buffer W1 (50 mM N-(2-hydroxyethyl) piperazine-N′-2-ethanesulfonic acid (HEPES) (pH 6.6), 140 mM NaCl, 3mM MgCl_2_, 20% (w/v) glycerol and 0.1% dodecyl maltoside). UV visible spectra were recorded at 20°C using a Hitachi U-3000 dual beam spectrophotometer. Visual pigments were bleached for 3 min using a 60 W standard light bulb equipped with a Kodak Wratten #3 filter at a distance of 20 cm. Data were analyzed using Sigmaplot software (Jandel Scientific, San Rafael, CA).

### Statistical analyses

To evaluate all 127 θ values, it is necessary to obtain the λ_max_ values of the 60 structurally unstable mutant pigments. Take AncBoreotheria S1 with a single mutation T52F as an example. The T52F mutant is nonfunctional and, consequently, “cannot” shift the λ_max_ value of AncBoreotheria S1. In fact, when T52F is introduced into mouse S1, the mutant pigment is functional and does not cause any λ_max_-shift [10]. Therefore, 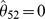 nm and 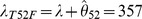
nm. AncBoreotheria S1 with F46T and A114G is another example. The single mutation analyses show that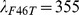 nm with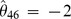 nm and 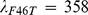
nm with 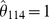
nm; when the two mutations are combined, they “cannot” shift the λ_max_ and, therefore, 
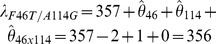
nm. This procedure and logic also can be justified by conducting the analysis on a data set that contained only the λ_max_ values for structurally stable pigments. In this reduced analysis not all effects and interactions can be estimated, but the corresponding θ values obtained from the two methods are identical.

The λ_max_ values of the 127 SWS1 mutant pigments were expressed as that of AncBoreotheria S1 (λ) plus the effects of the appropriate single and multiple amino acid changes on the λ_max_-shift (denoted by a sum of θ values). These θ values were estimated by solving a total of 128 simultaneous linear equations with the ancestral λ and 127 mutant values 
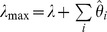
.

Estimation of effects and interactions of amino acid changes on the λ_max_ values were conducted using matrix algebra and linear statistical models [47]. The rows of the coefficient matrix C represent the sequences and the columns represent the effects and interaction to be estimated. The coefficients in the first column of C are all 1s indicating the ancestral sequence is included in all derived sequences. The elements of the remaining columns have the coefficients (0 or 1) for the individual effects and interactions present in the sequence designated by the row.

The parameters are designated in the column vector X, while column vector Y contains the observed λ_max_ values corresponding to the pigments. The simultaneous equations for the observed λ_max_ values are expressed in matrix algebra as:
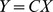



And the estimates of the effects and interactions are obtained by solving the equation for X, i.e., 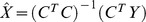


Since the estimates are linear functions of the observed λ_max_ values then the variance of each estimate is a function of the variances of the λ_max_ values and the coefficient associated with each parameter. For this analysis the variances for λ_max_ are assumed to be 1 and covariance 0. Given the variance-covariance matrix for Y is V then the standard errors of the estimates are on the diagonal of Se.
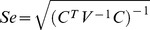



### Modeling of protein structures

The structures of AncBoreotheria S1 and mouse S1 pigments were obtained by 1) applying homology modeling (Modeller 9v7, www.salilab.org/modeller) to bovine rhodopsin (pdb code: 1U19), 2) adding the missing hydrogen atoms, water molecules, and 11-*cis*-retinal, and 3) optimizing them first at pure AMBER96 force field level (http://ambermd.org) and then using hybrid quantum mechanical/molecular mechanical (QM/MM) calculations in the ONIOM electronic embedding scheme (QM  =  B3LYP/6–31G*; MM  =  AMBER) [24]. The QM part of the calculations involves 11-*cis*-retinal with the covalently bound Schiff base (SB) nitrogen, or NH moiety, and E113 along with hydrogen link atoms, in which either the SB nitrogen or one of the carboxylic O atoms of the E113 is protonated, resulting retinal with protonated SB nitrogen (or PSBR) or retinal with unprotonated SB nitrogen (or SBR), respectively.

## Supporting Information

S1 Fig **Evolutionary trajectories with premature termination.** Each termination event due to unstable protein structure is indicated by a filled red square.
(EPS)

S2 Fig **All accessible evolutionary trajectories with seven amino acid substitutions in AncBoreotheria S1.** Six levels of λ_max_ values are color coded and the possibilities of whether or not a path includes a large phenotypic change (Δλ_max_>25 nm) are also indicated.
(EPS)

S3 Fig **The variances of Δλ_max_ values along an evolutionary path based on whether or not it contains Δλ_max_>30 nm.** The green and blue columns indicate paths contain Δλ_max_ <30 nm and Δλ_max_> 30 nm, respectively. The two red bars indicate 8 paths derived from Fig. 4, four have variances that range from (46.2–49.2) and four range from (85.9–89.9).
(EPS)

S4 Fig **The amino acid sequences of AncBoreotheria S1, pigment g and human S1.** Pigment g was reconstructed previously [21]. Dots indicate the identity of the amino acids with those of AncBoreotheria S1. The amino acids in red are taken from those of the SWS1 pigment of *Anolis carolinensis*, which is done to standardize the effects of variable amino acids at the N- and C-termini of SWS1 pigments on the λ_max_-shift. Seven transmembrane segments (orange shade) and seven critical amino acid residues (star) are indicated.
(EPS)

S1 Table **The amino acid changes, λ_max_ values and λ_max_-shifts caused by the amino acid changes in AncBoreotheria S1.**
(PDF)

S2 Table **The λ_max_-shifts caused by the single and multiple amino acid changes.**
(PDF)

S3 Table **T93P/A114G, F86L, F49L, S118T, F46T and T52F, in that order.**
(PDF)
