# Supplementary material for: Epistatic Adaptive Evolution of Human Color Vision
Source: PLoS Genet. 2014 Dec 18;10(12):e1004884. doi: 10.1371/journal.pgen.1004884 (PMC4270479; doi:10.1371/journal.pgen.1004884)
Supplement: S1 Table — The amino acid changes, λmax values and λmax-shifts caused by the amino acid changes in AncBoreotheria S1. (PDF) [file pgen.1004884.s005.pdf]

F46T	F49L	T52F	F86L	T93P	A114G	S118T	λ_{max} (nm)	$\Delta\lambda_{max}$ (nm)	F46T	F49L	T52F	F86L	T93P	A114G	S118T	λ_{max} (nm)	$\Delta\lambda_{max}$ (nm)
0	0	0	0	0	0	0	357		1	1	1	1	0	0	0	***357***	***0***
1	0	0	0	0	0	0	355	-2	1	1	1	0	1	0	0	***355***	***0***
0	1	0	0	0	0	0	354	-3	1	1	1	0	0	1	0	***357***	***0***
0	0	1	0	0	0	0	***357***	***0***	1	1	1	0	0	0	1	***359***	***0***
0	0	0	1	0	0	0	357	0	1	1	0	1	1	0	0	389	32
0	0	0	0	1	0	0	359	2	1	1	0	1	0	1	0	***357***	***0***
0	0	0	0	0	1	0	358	1	1	1	0	1	0	0	1	***358***	***0***
0	0	0	0	0	0	1	358	1	1	1	0	0	1	1	0	***361***	***0***
1	1	0	0	0	0	0	355	-2	1	1	0	0	1	0	1	358	1
1	0	1	0	0	0	0	356	-1	1	1	0	0	0	1	1	***357***	***0***
1	0	0	1	0	0	0	358	1	1	0	1	1	1	0	0	390	33
1	0	0	0	1	0	0	358	1	1	0	1	1	0	1	0	***360***	***0***
1	0	0	0	0	1	0	***356***	***0***	1	0	1	1	0	0	1	***361***	***0***
1	0	0	0	0	0	1	358	1	1	0	1	0	1	1	0	***357***	***0***
0	1	1	0	0	0	0	***354***	***0***	1	0	1	0	1	0	1	***357***	***0***
0	1	0	1	0	0	0	353	-4	1	0	1	0	0	1	1	**358**	**0**
0	1	0	0	1	0	0	356	-1	1	0	0	1	1	1	0	391	34
0	1	0	0	0	1	0	***355***	***0***	1	0	0	1	1	0	1	397	40
0	1	0	0	0	0	1	***355***	***0***	1	0	0	1	0	1	1	***362***	***0***
0	0	1	1	0	0	0	***357***	***0***	1	0	0	0	1	1	1	362	5
0	0	1	0	1	0	0	356	-1	0	1	1	1	1	0	0	396	39
0	0	1	0	0	1	0	***358***	***0***	0	1	1	1	0	1	0	***353***	***0***
0	0	1	0	0	0	1	***358***	***0***	0	1	1	1	0	0	1	***356***	***0***
0	0	0	1	1	0	0	377	20	0	1	1	0	1	1	0	***356***	***0***
0	0	0	1	0	1	0	357	0	0	1	1	0	1	0	1	***353***	***0***
0	0	0	1	0	0	1	360	1	0	1	1	0	0	1	1	***354***	***0***
0	0	0	0	1	1	0	361	4	0	1	0	1	1	1	0	385	28
0	0	0	0	1	0	1	359	2	0	1	0	1	1	0	1	403	46
0	0	0	0	0	1	1	357	0	0	1	0	1	0	1	1	***357***	***0***
1	1	1	0	0	0	0	***356***	***0***	0	1	0	0	1	1	1	363	6
1	1	0	1	0	0	0	356	-1	0	0	1	1	1	1	0	394	37
1	1	0	0	1	0	0	358	1	0	0	1	1	1	0	1	408	61
1	1	0	0	0	1	0	***356***	***0***	0	0	1	1	0	1	1	***361***	***0***
1	1	0	0	0	0	1	***358***	***0***	0	0	1	0	1	1	1	***360***	***0***
1	0	1	1	0	0	0	***359***	***0***	0	0	0	1	1	1	1	402	45
1	0	1	0	1	0	0	355	-2	1	1	1	1	1	0	0	394	37
1	0	1	0	0	1	0	***357***	***0***	1	1	1	1	0	1	0	***358***	***0***
1	0	1	0	0	0	1	***359***	***0***	1	1	1	1	0	0	1	***359***	***0***
1	0	0	1	1	0	0	386	29	1	1	1	0	1	1	0	***358***	***0***
1	0	0	1	0	1	0	359	2	1	1	1	0	1	0	1	***355***	***0***
1	0	0	1	0	0	1	360	3	1	1	1	0	0	1	1	***358***	***0***
1	0	0	0	1	1	0	360	3	1	1	0	1	1	1	0	394	37
1	0	0	0	1	0	1	360	3	1	1	0	1	1	0	1	401	44
1	0	0	0	0	1	1	***357***	***0***	1	1	0	1	0	1	1	369	12
0	1	1	1	0	0	0	***353***	***0***	1	1	0	0	1	1	1	***363***	***0***
0	1	1	0	1	0	0	***353***	***0***	1	0	1	1	1	1	0	395	38
0	1	1	0	0	1	0	***355***	***0***	1	0	1	1	1	0	1	394	37
0	1	1	0	0	0	1	***355***	***0***	1	0	1	1	0	1	1	363	6
0	1	0	1	1	0	0	388	31	1	0	1	0	1	1	1	***359***	***0***
0	1	0	1	0	1	0	***353***	***0***	1	0	0	1	1	1	1	402	45
0	1	0	1	0	0	1	***356***	***0***	0	1	1	1	1	1	0	394	37
0	1	0	0	1	1	0	359	2	0	1	1	1	1	0	1	406	49
0	1	0	0	1	0	1	356	-1	0	0	1	1	0	1	1	***357***	***0***
0	1	0	0	0	1	1	***354***	***0***	0	1	1	0	1	1	1	***360***	***0***
0	0	1	1	1	0	0	388	31	0	1	0	1	1	1	1	399	42
0	0	1	1	0	1	0	***357***	***0***	0	0	1	1	1	1	1	410	53
0	0	1	1	0	0	1	***360***	***0***	1	1	1	1	1	1	0	393	36
0	0	1	0	1	1	0	***358***	***0***	1	1	1	1	1	0	1	407	50
0	0	1	0	1	0	1	***356***	***0***	1	1	1	1	0	1	1	***370***	***0***
0	0	1	0	0	1	1	***357***	***0***	1	1	1	0	1	1	1	***360***	***0***
0	0	0	1	1	1	0	381	24	1	1	0	1	1	1	1	404	47
0	0	0	1	1	0	1	399	42	1	0	1	1	1	1	1	407	50
0	0	0	1	0	1	1	361	4	0	1	1	1	1	1	1	408	51
0	0	0	0	1	1	1	363	6	1	1	1	1	1	1	1	411	54

Numbers 0 and 1 represent the amino acids of AncBoreotheria S1 and human S1, respectively. Letters in bold italics indicate that some λ_{max} values involved are inferred from structurally unstable pigments.
