# Supplementary material for: Epistatic Adaptive Evolution of Human Color Vision
Source: PLoS Genet. 2014 Dec 18;10(12):e1004884. doi: 10.1371/journal.pgen.1004884 (PMC4270479; doi:10.1371/journal.pgen.1004884)
Supplement: S2 Table — The λmax-shifts caused by the single and multiple amino acid changes. (PDF) [file pgen.1004884.s006.pdf]

Mutation	θ (nm) ± se	Mutation	θ (nm) ± se
46	-2 ± 1.4	***46 x 49 x 52 x 93***	***0***
49	-3 ± 1.4	***46 x 49 x 52 x 114***	***0***
52	***0***	***46 x 49 x 52 x 118***	***0***
86	0 ± 1.4	46 x 49 x 86 x 93	-10 ± 4.0
93	2 ± 1.4	***46 x 49 x 86 x 114***	***0***
114	1 ± 1.4	***46 x 49 x 86 x 118***	***0***
118	1 ± 1.4	***46 x 49 x 93 x 114***	***0***
46 x 49	3 ± 2.0	46 x 49 x 93 x 118	-2 ± 4.0
46 x 52	1 ± 2.0	***46 x 49 x 114 x 118***	***0***
46 x 86	3 ± 2.0	46 x 52 x 86 x 93	-7 ± 4.0
46 x 93	1 ± 2.0	***46 x 52 x 86 x 114***	***0***
46 x 114	***0***	***46 x 52 x 86 x 118***	***0***
46 x 118	2 ± 2.0	***46 x 52 x 93 x 114***	***0***
49 x 52	***0***	***46 x 52 x 93 x 118***	***0***
49 x 86	-1 ± 2.0	***46 x 52 x 114 x 118***	***0***
49 x 93	0 ± 2.0	***46 x 86 x 93 x 114***	***0***
49 x 114	***0***	46 x 86 x 93 x 118	-10 ± 4.0
49 x 118	***0***	***46 x 86 x 114 x 118***	***0***
52 x 86	***0***	46 x 93 x 114 x 118	-2 ± 4.0
52 x 93	-3 ± 2.0	49 x 52 x 86 x 93	-3 ± 4.0
52 x 114	***0***	***49 x 52 x 86 x 114***	***0***
52 x 118	***0***	***49 x 52 x 86 x 118***	***0***
86 x 93	18 ± 2.0	***49 x 52 x 93 x 114***	***0***
86 x 114	-1 ± 2.0	***49 x 52 x 93 x 118***	***0***
86 x 118	2 ± 2.0	***49 x 52 x 114 x 118***	***0***
93 x 114	1 ± 2.0	49 x 86 x 93 x 114	-8 ± 4.0
93 x 118	-1 ± 2.0	49 x 86 x 93 x 118	-7 ± 4.0
114 x 118	-2 ± 2.0	***49 x 86 x 114 x 118***	***0***
46 x 49 x 52	***0***	49 x 93 x 114 x 118	2 ± 4.0
46 x 49 x 86	-1 ± 2.8	52 x 86 x 93 x 114	2 ± 4.0
46 x 49 x 93	0 ± 2.8	52 x 86 x 93 x 118	-2 ± 4.0
46 x 49 x 114	***0***	***52 x 86 x 114 x 118***	***0***
46 x 49 x 118	***0***	***52 x 93 x 114 x 118***	***0***
46 x 52 x 86	***0***	86 x 93 x 114 x 118	-6 ± 4.0
46 x 52 x 93	-1 ± 2.8	46 x 49 x 52 x 86 x 93	4 ± 5.7
46 x 52 x 114	***0***	***46 x 49 x 52 x 86 x 114***	***0***
46 x 52 x 118	***0***	***46 x 49 x 52 x 86 x 118***	***0***
46 x 86 x 93	7 ± 2.8	***46 x 49 x 52 x 93 x 114***	***0***
46 x 86 x 114	1 ± 2.8	***46 x 49 x 52 x 93 x 118***	***0***
46 x 86 x 118	-3 ± 2.8	***46 x 49 x 52 x 114 x 118***	***0***
46 x 93 x 114	0 ± 2.8	46 x 49 x 86 x 93 x 114	7 ± 5.7
46 x 93 x 118	0 ± 2.8	46 x 49 x 86 x 93 x 118	10 ± 5.7
46 x 114 x 118	***0***	46 x 49 x 86 x 114 x 118	9 ± 5.7
49 x 52 x 86	***0***	***46 x 49 x 93 x 114 x 118***	***0***
49 x 52 x 93	***0***	46 x 52 x 86 x 93 x 114	-2 ± 5.7
49 x 52 x 114	***0***	46 x 52 x 86 x 93 x 118	-5 ± 5.7
49 x 52 x 118	***0***	46 x 52 x 86 x 114 x 118	0 ± 5.7
49 x 86 x 93	15 ± 2.8	***46 x 52 x 93 x 114 x 118***	***0***
49 x 86 x 114	***0***	46 x 86 x 93 x 114 x 118	3 ± 5.7
49 x 86 x 118	***0***	49 x 52 x 86 x 93 x 114	-1 ± 5.7
49 x 93 x 114	1 ± 2.8	49 x 52 x 86 x 93 x 118	-3 ± 5.7
49 x 93 x 118	0 ± 2.8	***49 x 52 x 86 x 114 x 118***	***0***
49 x 114 x 118	***0***	***49 x 52 x 93 x 114 x 118***	***0***
52 x 86 x 93	14 ± 2.8	49 x 86 x 93 x 114 x 118	-2 ± 5.7
52 x 86 x 114	***0***	52 x 86 x 93 x 114 x 118	-3 ± 5.7
52 x 86 x 118	***0***	46 x 49 x 52 x 86 x 93 x 114	-5 ± 8.0
52 x 93 x 114	***0***	46 x 49 x 52 x 86 x 93 x 118	11 ± 8.0
52 x 93 x 118	***0***	***46 x 49 x 52 x 86 x 114 x 118***	***0***
52 x 114 x 118	***0***	***46 x 49 x 52 x 93 x 114 x 118***	***0***
86 x 93 x 114	3 ± 2.8	46 x 49 x 86 x 93 x 114 x 118	-11 ± 8.0
86 x 93 x 118	20 ± 2.8	46 x 52 x 86 x 93 x 114 x 118	11 ± 8.0
86 x 114 x 118	3 ± 2.8	49 x 52 x 86 x 93 x 114 x 118	8 ± 8.0
93 x 114 x 118	4 ± 2.8	46 x 49 x 52 x 86 x 93 x 114 x 118	-9 ± 11.3
46 x 49 x 52 x 86	***0***		

Letters 46, 49, 52, 86, 93, 114 and 118 indicate F46T, F49L, T52F, F86L, T93P, A114G and S118T, respectively.
