# Supplementary material for: Epistatic Adaptive Evolution of Human Color Vision
Source: PLoS Genet. 2014 Dec 18;10(12):e1004884. doi: 10.1371/journal.pgen.1004884 (PMC4270479; doi:10.1371/journal.pgen.1004884)
Supplement: S3 Table — T93P/A114G, F86L, F49L, S118T, F46T and T52F, in that order. (PDF) [file pgen.1004884.s007.pdf]

Mutation	$\Delta\lambda_{max}$ (nm)	λ_{max} (nm) Exp.	Obs.
T93P/A114G	θ_{93x114} (1)	358	361
F86L	θ_{86x93} (18), θ_{86x114} (-1), $\theta_{86x93x114}$ (3)	378	381
F49L	θ_{49x86} (-1), θ_{49x93} (0), $\theta_{49x86x93}$ (15), θ_{49x114}^{*} + $\theta_{49x93x114}$ (1), $\theta_{49x86x114}^{*}$ + $\theta_{49x86x93x114}$ (-8)	385	385
S118T	θ_{86x118} (2), θ_{93x118} (-1), $\theta_{114x118}$ (-2), $\theta_{86x93x118}$ (20), $\theta_{86x114x118}$ (3), $\theta_{93x114x118}$ (4), $\theta_{86x93x114x118}$ (-6), θ_{49x118}^{*} + $\theta_{49x93x118}$ (0), $\theta_{49x86x118}^{*}$ + $\theta_{49x86x93x118}$ (-7), $\theta_{49x114x118}^{*}$ + $\theta_{49x93x114x118}$ (2), $\theta_{49x86x114x118}^{*}$ + $\theta_{49x86x93x114x118}$ (-2)	398	399
F46T	θ_{46x49} (3), θ_{46x86} (3), θ_{46x93} (1), θ_{46x118} (2), $\theta_{46x49x86}$ (-1), $\theta_{46x49x93}$ (0), $\theta_{46x86x93}$ (7), $\theta_{46x86x118}$ (-3), $\theta_{46x93x118}$ (0), $\theta_{46x49x86x93}$ (-10), $\theta_{46x86x93x118}$ (-10), θ_{46x114}^{*} + $\theta_{46x93x114}$ (0), $\theta_{46x86x114}^{*}$ + $\theta_{46x86x93x114}$ (1), $\theta_{46x49x114}^{*}$ + $\theta_{46x49x86x114}^{*}$ + $\theta_{46x49x93x114}^{*}$ + $\theta_{46x49x86x93x114}$ (7), $\theta_{46x114x118}^{*}$ + $\theta_{46x93x114x118}$ (-2), $\theta_{46x49x118}^{*}$ + $\theta_{46x49x93x118}$ (-2), $\theta_{46x49x86x118}^{*}$ + $\theta_{46x49x86x93x118}$ (10), $\theta_{46x49x114x118}^{*}$ + $\theta_{46x49x86x114x118}^{*}$ + $\theta_{46x49x93x114x118}^{*}$ + $\theta_{46x49x86x93x114x118}$ (-2), $\theta_{46x86x114x118}^{*}$ + $\theta_{46x86x93x114x118}$ (3)	405	404
T52F	θ_{46x52} (1), θ_{52x93} (-3), $\theta_{46x52x93}$ (-1), $\theta_{46x52x86}^{*}$ + $\theta_{46x52x86x93}$ (-7), θ_{49x52}^{*} + $\theta_{49x52x86}^{*}$ + $\theta_{49x52x93}^{*}$ + $\theta_{49x52x86x93}$ (-3), θ_{52x86}^{*} + $\theta_{52x86x93}$ (14), θ_{52x114}^{*} + $\theta_{52x86x114}^{*}$ + $\theta_{52x93x114}^{*}$ + $\theta_{52x86x93x114}$ (2), θ_{52x118}^{*} + $\theta_{52x86x118}^{*}$ + $\theta_{52x93x118}^{*}$ + $\theta_{52x86x93x118}$ (-2), $\theta_{46x52x114}^{*}$ + $\theta_{46x52x86x114}^{*}$ + $\theta_{46x52x93x114}^{*}$ + $\theta_{46x52x86x93x114}$ (-2), $\theta_{46x52x118}^{*}$ + $\theta_{46x52x86x118}^{*}$ + $\theta_{46x52x93x118}^{*}$ + $\theta_{46x52x86x93x118}$ (-5), $\theta_{49x52x114}^{*}$ + $\theta_{49x52x86x114}^{*}$ + $\theta_{49x52x93x114}^{*}$ + $\theta_{49x52x86x93x114}$ (-1), $\theta_{49x52x118}^{*}$ + $\theta_{49x52x86x118}^{*}$ + $\theta_{49x52x93x118}^{*}$ + $\theta_{49x52x86x93x118}$ (-3), $\theta_{52x114x118}^{*}$ + $\theta_{52x86x114x118}^{*}$ + $\theta_{52x93x114x118}^{*}$ + $\theta_{52x86x93x114x118}$ (-3), $\theta_{46x52x114x118}^{*}$ + $\theta_{46x52x86x114x118}^{*}$ + $\theta_{46x52x93x114x118}^{*}$ + $\theta_{46x52x86x93x114x118}$ (11), $\theta_{49x52x114x118}^{*}$ + $\theta_{49x52x86x114x118}^{*}$ + $\theta_{49x52x93x114x118}^{*}$ + $\theta_{49x52x86x93x114x118}$ (8), $q_{46x49x52}^{*}$ + $\theta_{46x49x52x86}^{*}$ + $\theta_{46x49x52x93}^{*}$ + $\theta_{46x49x52x86x93}$ (4), $\theta_{46x49x52x114}^{*}$ + $\theta_{46x49x52x86x114}^{*}$ + $\theta_{46x49x52x93x114}^{*}$ + $\theta_{46x49x52x86x93x114}$ (-5), $\theta_{46x49x52x118}^{*}$ + $\theta_{46x49x52x86x118}^{*}$ + $\theta_{46x49x52x93x118}^{*}$ + $\theta_{46x49x52x86x93x118}$ (11), $\theta_{46x49x52x114x118}^{*}$ + $\theta_{46x49x52x86x114x118}^{*}$ + $\theta_{46x49x52x93x114x118}^{*}$ + $\theta_{46x49x52x86x93x114x118}$ (-9)	412	411

Stars (*) indicate that θ values cannot be determined individually. Exp: Expected λ_{max} values based on the epistatic effects. Obs: Observed λ_{max} values from mutant pigments.
